# Rxiv-Maker – an automated template engine for streamlined scientific publications

**DOI:** 10.1242/jcs.265183

**Published:** 2026-10-12

**Authors:** Bruno M. Saraiva, Rita Carlota, António D. Brito, Iván Hidalgo-Cenalmor, Guillaume Jacquemet, Ricardo Henriques

**Affiliations:** ^1^ Instituto de Tecnologia Química e Biológica António Xavier, Universidade Nova de Lisboa, 2780-157 Oeiras, Portugal; ^2^ Turku Bioscience Centre, University of Turku and Åbo Akademi University, 20520 Turku, Finland; ^3^ Faculty of Science and Engineering, Cell Biology, Åbo Akademi University, 20520 Turku, Finland; ^4^ InFLAMES Research Flagship Center, University of Turku and Åbo Akademi University, 20520 Turku, Finland; ^5^ Foundation for the Finnish Cancer Institute, Tukholmankatu 8, 00014 Helsinki, Finland

**Keywords:** Preprints, Scientific publishing, Reproducibility, Open-source software, Markdown, Bioimage analysis

## Abstract

Preprints have transformed how biologists share results; yet, how well a preprint reads still depends on formatting, a task that has shifted from publishers to authors. Cell biology has also become increasingly quantitative, with conclusions resting on multi-step image analysis, for which figures, values and statistics are revised repeatedly across a project. Keeping a well-formatted manuscript in step with the latest analysis is tedious and error prone. We introduce Rxiv-Maker, an open-source framework that lets authors write in simple Markdown and produce a formatted article, as a PDF or an editable Word document, with consistent styling, automatic figure and citation numbering, and reliable cross-referencing. For quantitative work, it can optionally regenerate figures and recompute reported values directly from the data, so the text stays consistent with the analysis. Built on familiar tools such as Git and Visual Studio Code, it gives a transparent, collaborative path from analysis to a submission-ready preprint. Researchers have already used Rxiv-Maker for cell biology studies spanning bacterial cell cycle analysis, filopodia proteomics and zebrafish live imaging, showing that it supports reproducible reporting of genuine biological findings.

## Teaser

**Summary:** Rxiv-Maker is an open-source framework that lets researchers draft manuscripts in simple Markdown to automatically generate formatted preprints and Word documents.

## INTRODUCTION

Scientific publishing has been reshaped by the rise of preprint servers, which allow researchers to share their findings quickly and openly across many platforms (Beck et al., 2020 preprint; Fraser et al., 2020; Levchenko et al., 2024) (Figs S1 and S2). This has improved scientific communication, but it has also shifted many practical tasks, such as layout, formatting, file packaging and meeting the technical requirements of each platform, from publishers onto researchers (Vale, 2015; Tennant et al., 2016; Lin et al., 2020). For example, in disciplines such as bioimage analysis, in which findings are built upon complex computational pipelines and conclusions often depend on iterative parameter adjustments, ensuring that a well-formatted document stays consistent with the latest data remains a challenge for many scientists (Rubens et al., 2020; Hidalgo-Cenalmor et al., 2024). This is a familiar situation in cell biology, in which a typical study couples quantitative microscopy with segmentation, tracking and statistical analysis, and the reported figures and numbers are revised many times before submission.

To address these challenges, we developed Rxiv-Maker, a framework designed to simplify and automate the creation of scientific manuscripts. It allows authors to focus on their writing in plain text while the engine handles layouts, citations and figure placements. For researchers who need strict consistency, it can optionally act as a self-updating manuscript by executing embedded Python or R scripts during compilation, so the final document reflects the most up-to-date results. The framework is designed in a way that most of the work happens directly on the researcher's computer. This makes writing and editing fast and responsive. It also works smoothly with Git, the version control system that records changes to files, and with Git-based hosting platforms, such as GitHub. This helps researchers keep a clear record of edits, improves transparency, and makes it easier for multiple people to collaborate on the same project (Ram, 2013; Perez-Riverol et al., 2016). The architecture and processing pipeline are illustrated in Fig. 1, and a visual example of the files with the generated PDF file can be seen in Fig. 2. A tutorial on the project documentation site (https://rxiv-maker.henriqueslab.org/getting-started/first-manuscript/) guides new users through their first manuscript.

In summary, Rxiv-Maker enables authors to write manuscripts in Markdown and converts them into formatted documents without requiring authoring directly in LaTeX. It focuses on features that improve readability and workflow, including handling of figures and captions, automatic numbering and cross-references, and citation management. This helps keep manuscripts consistent throughout drafting, revision and submission, while ensuring that figures, graphs and tables stay updated with the latest analyses.

## RESULTS

### From text to a manuscript – Markdown and document structure

In life sciences, LaTeX remains one of the most reliable systems for ensuring consistent layouts, accurate equation rendering, controlled figure placement and reliable cross-referencing when preparing manuscripts. However, syntax errors can interrupt compilation, and collaboration can be challenging when co-authors have varying levels of familiarity with the markup. Rxiv-Maker addresses these formatting bottlenecks by allowing researchers to separate drafting from typesetting. Authors write manuscripts in Markdown that is automatically converted into PDF and Word outputs.

**Fig. 1.**
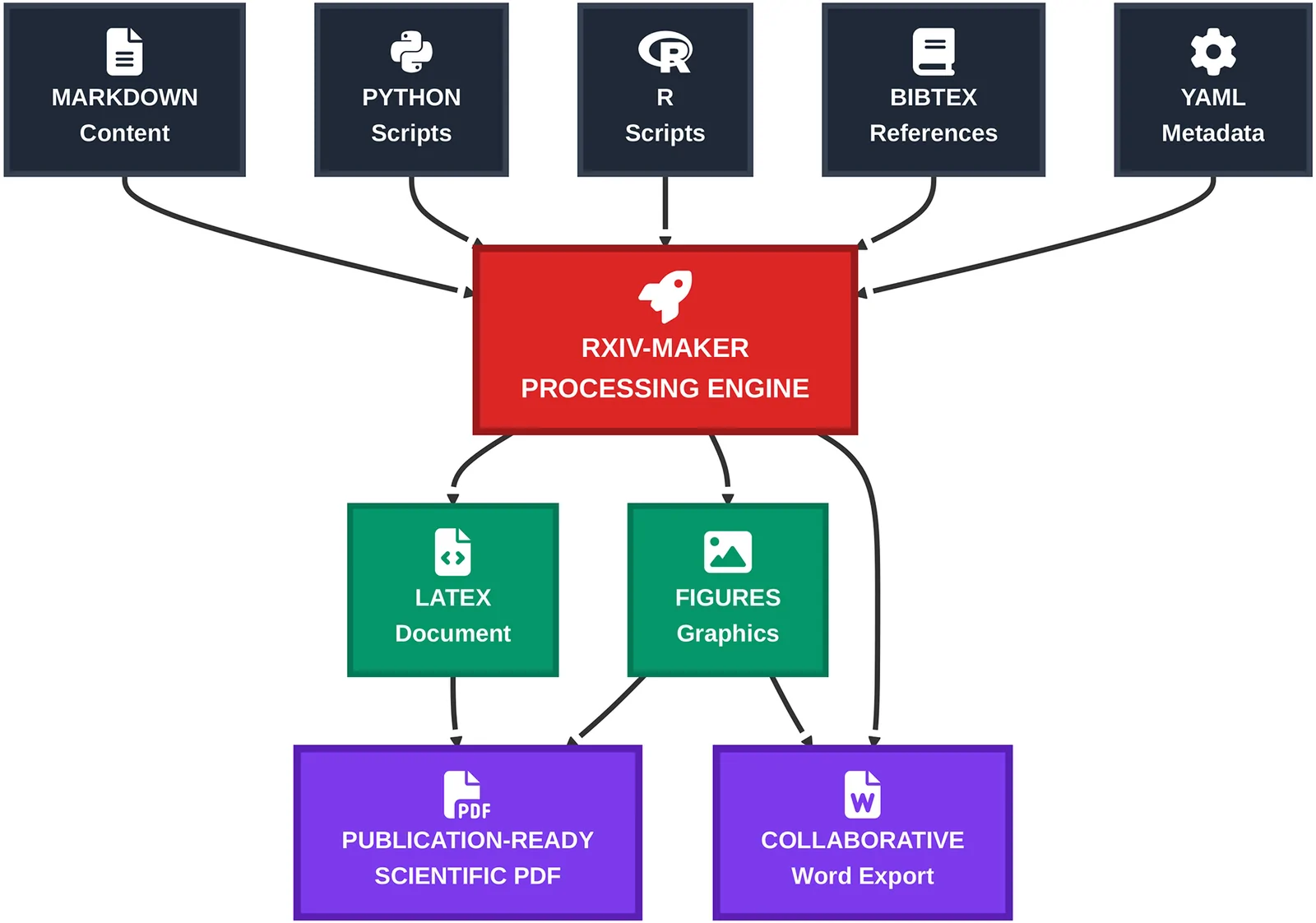
**System architecture.** Rxiv-Maker integrates Markdown content, YAML metadata, executable scripts and bibliographies through a processing engine that combines local execution with LaTeX PDF compilation or Word (.docx) generation.

**Fig. 2.**
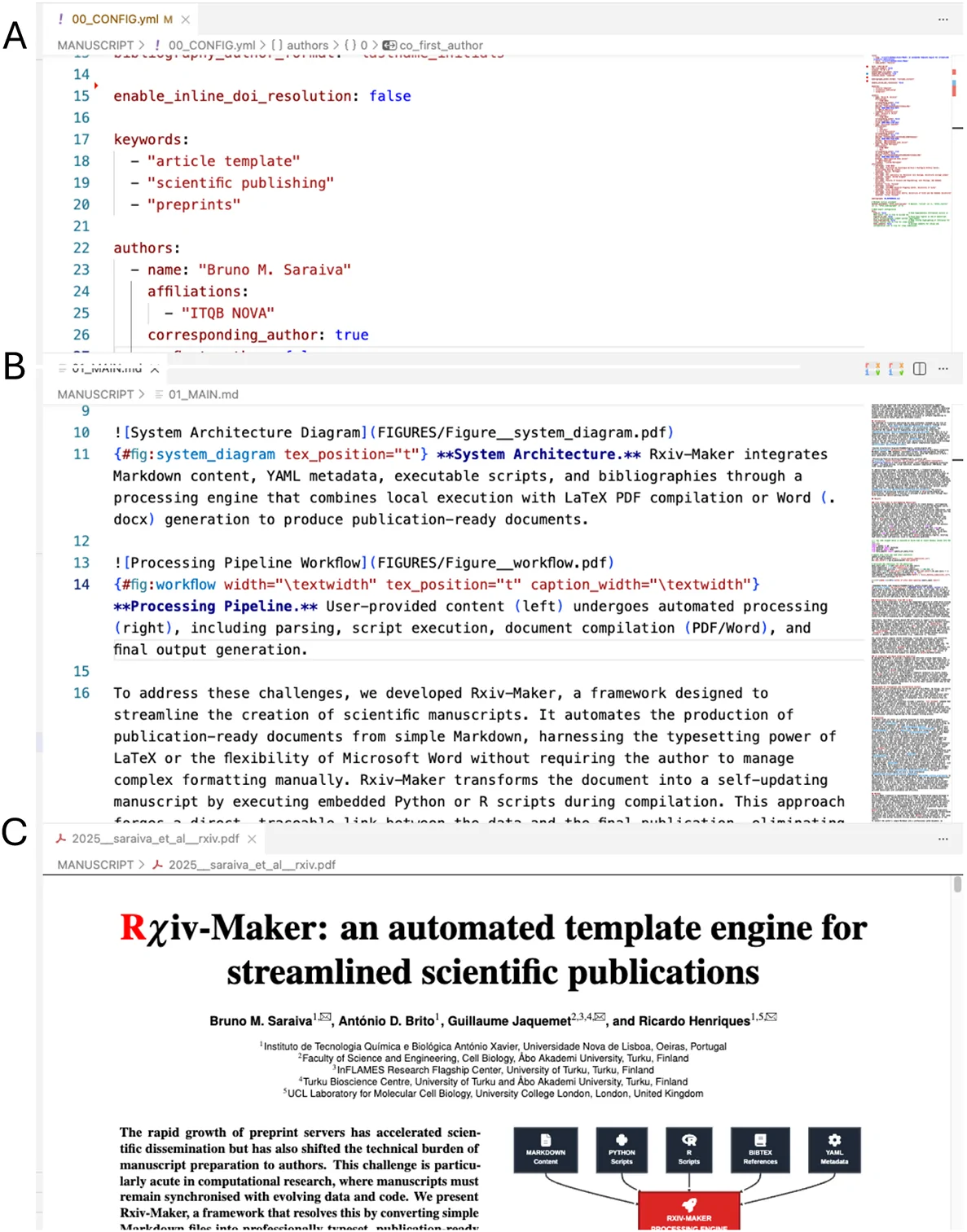
**From YAML and Markdown files to a typeset PDF.** (A) Example config.yml file illustrating the structured specification of manuscript metadata and build parameters. This configuration file defines the global document settings and ensures reproducible manuscript generation. (B) Main manuscript source file written in Markdown (sections, figures, references and cross-references) in a human-readable and version-controllable format. (C) Final compiled PDF generated by Rxiv-Maker from the configuration and Markdown sources.

Markdown is a widely used syntax that is easy to read and sufficiently expressive to capture the structure of a research article without requiring authors to think in terms of code. Instead, it uses simple characters (such as *, # and @) to add meaning to the text. This is similar to what happens, for instance, in WhatsApp or Discord, where surrounding text with asterisks applies formatting such as bold or italic, depending on the platform. Authors do not need to adopt a new writing style: manuscripts can be drafted in any text editor, or even a word processor, and then copied into the Markdown source with minimal adjustments, such as adding section headings, figure callouts and citations, before generating the final PDF. These commands are straightforward to use and are documented in full on the project website, which carries installation instructions, a walkthrough for a first manuscript, worked examples, and a reference for every supported element (https://rxiv-maker.henriqueslab.org). Researchers who want to try the workflow before installing anything can open a prepared Google Colab notebook and build an example manuscript in the browser (https://colab.research.google.com/github/HenriquesLab/rxiv-maker/blob/main/notebooks/rxivmakercolab.ipynb), which lowers the barrier for colleagues who are wary of a local installation.

When a manuscript is compiled, Rxiv-Maker converts the Markdown into a typeset document. It first safeguards the elements most easily disrupted, such as equations, code and references, and then translates the text and formatting into the underlying typesetting instructions, covering the full range of elements found in a research article.

To support efficient iteration, Rxiv-Maker avoids re-running unchanged processing steps, such as previously generated figures. A companion extension for Visual Studio Code (Fig. S3) provides syntax highlighting, autocompletion for citation keys and cross-references, and real-time validation of the configuration of the manuscript. Bringing authoring and validation into one familiar environment lets researchers focus on content while the tool handles typesetting.

Within Rxiv-Maker, a manuscript is organised as a structured folder containing a main manuscript file, an optional supplementary text file, a bibliography file and a figures directory (see Materials and Methods). These components are compiled to generate the final PDF (Fig. 1). This ‘manuscript-as-a-folder’ structure was chosen because it is transparent, easy to share with collaborators and compatible with version control.

To keep everything organised and avoid duplicating information across files and output formats, the manuscript details are centralised in a single plain-text configuration file. This file is the definitive source for the title, author list, affiliations, corresponding author details and formatting options (such as citation style or line numbering), so they stay consistent across every output and submission (Fig. 2).

### Advanced integration – keeping figures and numbers in step with the analysis

Beyond producing a well-formatted article, Rxiv-Maker offers an advanced, optional capability for quantitative, analysis-heavy work: keeping a manuscript in step with the data behind it. This functionality enables the direct execution of analysis scripts during compilation, with the results (whether statistical values, tables or figures) directly injected into the text. This process eliminates transcription errors when, for example, copying a value from a terminal or another program into the manuscript (Perkel, 2022).

For results that change often during revisions, we adopted a lightweight ‘compute once, reuse everywhere’ approach: authors can include a short Python snippet that runs a small analysis when the document is compiled (for example, loading a table of segmented cells and calculating the sample size or summary statistics), and the resulting values are inserted straight into the text or figure legends. The analysis runs inside the folder of the manuscript itself, so that it can locate the data files for the project directly. This is the same pattern a cell biologist would use to keep a reported cell count, a mean fluorescence intensity or a migration speed synchronised with the underlying measurements as an analysis is refined. Fig. 3 shows a worked example embedded in the manuscript, where the values are computed as the document is built and placed into the narrative.

**Fig. 3.**
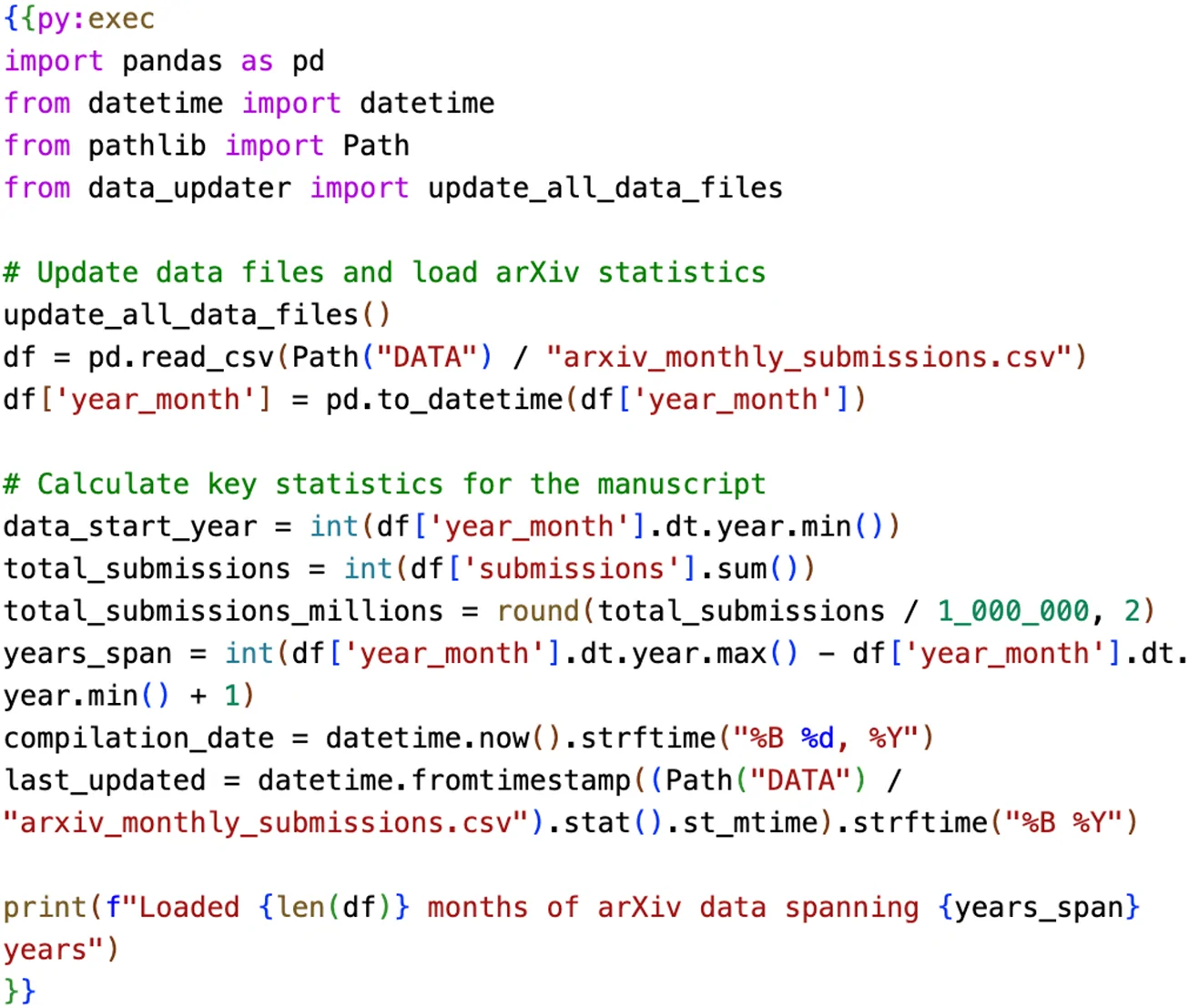
**Embedded Python for dynamic content.** This figure shows a Python script embedded in the source code of the manuscript. The script is executed at build time to compute data attributes (e.g. total submissions and span of years) and inject the resulting values directly into the text. This process, rendered here with syntax highlighting from the Rxiv-Maker Visual Studio Code extension, eliminates manual transcription errors and ensures that the text remains synchronised with the source data.

Authors can also place figure-generating code directly in the figures folder of the manuscript (for example, Python, R, or Mermaid, a text-based diagramming tool). Rxiv-Maker runs these files automatically to produce the figures used in the manuscript.

This approach facilitates synchronisation between evolving analyses and the manuscript, ensuring that figures remain up to date. At the same time, it accommodates collaborators without programming expertise, who can work with the manuscript using static figures and plain text where appropriate.

This capability extends to all forms of programmatic figure generation, as detailed in the Materials and Methods, so that programmatically generated figures and numerical values can be reproducibly regenerated and kept in step with the underlying data (Fig. S1).

### Proof of principle – adoption in biological research

Rxiv-Maker is already being used to prepare quantitative cell biology studies, evidence that the framework supports the reporting of genuine biological findings and not only method demonstrations. For instance, mAIcrobe, a framework for high-throughput bacterial image analysis, was written with Rxiv-Maker and used to quantify antibiotic-induced phenotypes in *Escherichia coli* and to identify cell cycle defects in *Staphylococcus aureus* DnaA mutants (Brito et al., 2026). In cell migration research, a filopodome proteomics study prepared with the framework identified the chaperonin subunit CCT8 as a MYO10 interactor required for normal filopodia function (Popović et al., 2026), and a related discovery study combined super-resolution live imaging with volume electron microscopy to show that epithelial filopodia can penetrate neighbouring cells and drive trans-endocytosis, identifying filopodia as exchange organelles between cells (Grobe et al., 2026 preprint). A developmental biology study used the framework to report a 3D-printed mould for live imaging of zebrafish embryos (Rivera Pineda et al., 2026). It has also been used to present new imaging methodology, including NucleiSky, which registers microscopy data across modalities and scales by treating the spatial arrangement of nuclei as an intrinsic biological fingerprint (Hidalgo-Cenalmor et al., 2026). Uptake also extends beyond the developers of the framework – an independent genomics group used Rxiv-Maker to prepare KaryoScope, an alignment-free genome-annotation method applied to Human Pangenome Reference Consortium assemblies (Ranallo-Benavidez et al., 2026 preprint). Spanning bacterial cell biology, cell migration, developmental biology and genome analysis, these studies show that Rxiv-Maker can carry a quantitative biological project from analysis through to a submission-ready manuscript.

### Collaboration across formats and reproducible versioning

Scientific manuscripts are rarely written alone, and co-authors or journals often require editable formats. Rxiv-Maker therefore extends beyond PDF generation: a dedicated Word export engine (see Materials and Methods) produces .docx files from the same Markdown source, and its integration with Git provides transparent versioning. Authors can generate annotated ‘diff’ PDFs to visualise and compare textual changes across manuscript versions or relative to specific milestones, for example, submission versus revision, echoing the familiar ‘Track changes’ workflow. The same comparison can be written to a Word file with the changes highlighted, which is the form most journals ask for when they request a marked copy alongside the clean one. Together, transparent versioning and rapid local iteration create a fully auditable history from the inception of a project to its final publication.

Running the same manuscript on another machine takes more than sharing the text, because a document that regenerates its own figures also depends on the versions of Python, R and LaTeX that produced them. Rxiv-Maker therefore publishes a container image that pins the entire build environment (see Materials and Methods). A collaborator who builds the manuscript inside that image provides a consistent, pinned build environment across supported systems, without installing a LaTeX distribution or matching package versions by hand. The image can also be archived with the paper, so the build still runs years later, when the original toolchain may no longer be installable (Boettiger, 2015; Gomes et al., 2018). This matters most for long projects and large collaborations, in which authors join at different times and on different systems.

### Submission-ready outputs

Before a manuscript leaves the author's computer, Rxiv-Maker runs checks that flag common problems early, such as missing figures, broken cross-references, and citation or bibliography errors. These checks can also be set to run automatically each time the manuscript is updated.

Using the same manuscript sources and YAML configuration file, Rxiv-Maker then generates submission-ready packages tailored for typical preprint workflows (Fig. 4). arXiv and bioRxiv impose different requirements: arXiv requires all LaTeX source files, figures in specific formats, bibliography files and style definitions, whereas bioRxiv requires compiled PDFs and individual figure files. Assembling these packages by hand is error prone and time consuming, requiring authors to collect and validate dozens of files while meeting platform-specific naming and format conventions.

**Fig. 4.**
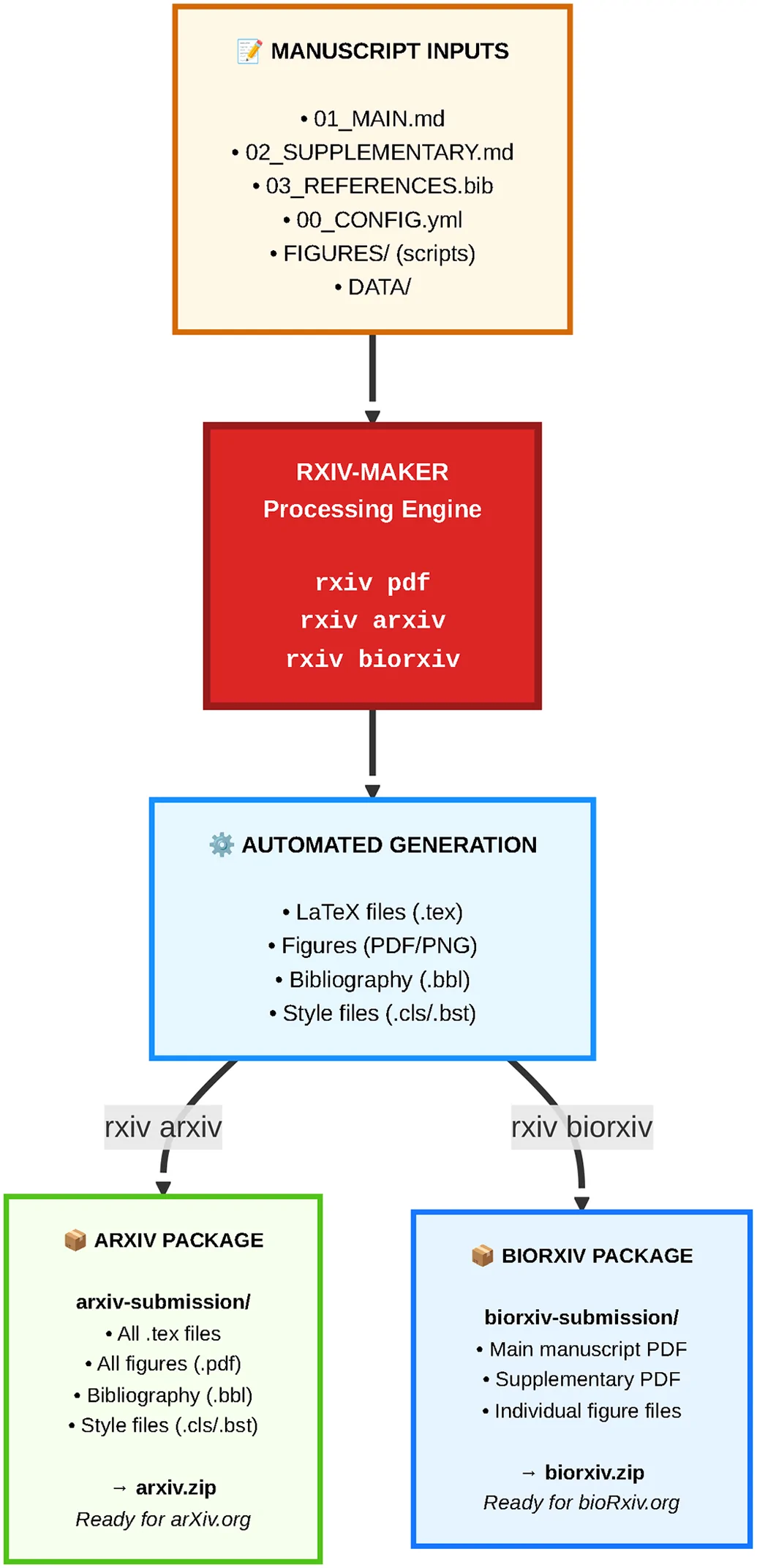
**Automated generation of submission-ready packages.** Rxiv-Maker transforms manuscript inputs (Markdown content, supplementary information, references, configuration, figures and data) through its processing engine to generate two distinct submission packages. The arXiv package contains all LaTeX source files (.tex), figures in PDF format, formatted bibliography (.bbl) and style files, bundled as arxiv.zip for direct upload to arXiv.org. The bioRxiv package contains compiled manuscript and supplementary PDFs along with individual figure files, bundled as biorxiv.zip for direct upload to bioRxiv.org. Both packages are generated with single commands (‘rxiv arxiv’ and ‘rxiv biorxiv’), eliminating manual file collection and ensuring compliance with platform-specific submission requirements.

This automation saves time and reduces the risk of desk rejection due to technical formatting issues, allowing researchers to focus on their science instead of submission logistics. By streamlining the path from manuscript to preprint, Rxiv-Maker removes a barrier in the open-science workflow and encourages rapid, compliant dissemination of research findings (Fig. 4).

## DISCUSSION

Rxiv-Maker sits alongside a growing set of tools for scientific publishing. Collaborative web-based editors like Overleaf (https://www.overleaf.com/) support team-based LaTeX writing but leave open the challenge of keeping a manuscript synchronised with external data and analysis code. Rxiv-Maker aligns more closely with platforms such as Manubot (Himmelstein et al., 2019), Jupyter Book (Executable Books Community; doi:10.5281/zenodo.4539666) and Quarto (https://quarto.org/), which also integrate code and narrative, often building on notebook-based formats (Kluyver et al., 2016). Rxiv-Maker is focused specifically on producing manuscripts and preprints without requiring programming expertise beyond basic Markdown syntax. Beyond preprint distribution, Rxiv-Maker supports the production of manuscripts suitable for submission to peer-reviewed journals, generating PDFs alongside clean LaTeX source code that is widely accepted prior to journal-specific formatting. Rxiv-Maker covers the path from initial draft through preprint distribution to journal submission. In comparison with other tools (Table S2), Rxiv-Maker combines integration with Git-based workflows and an optional capability that re-runs only changed analyses during compilation. We also note other modern typesetting systems such as Typst (https://typst.app/) and frameworks such as Bookdown (Xie, 2016), which offer alternative paradigms for scientific document creation.

We also note the limitations of the framework. Rxiv-Maker is not a real-time collaborative ‘what you see is what you get’ or WYSIWYG editor such as Google Docs or Overleaf; co-authors review through Word exports or version-control branches. It does not replace graphical layout software such as Adobe Illustrator, PowerPoint or BioRender for complex multi-panel biological diagrams, which must still be created manually and included as static image files. Local execution requires familiarity with command line tools or terminal environments, although the Google Colab workflow provides an alternative for authors who prefer a browser-based setup. Finally, code execution during compilation is opt-in and restricted to local scripts, so security and computational dependencies remain the responsibility of the author.

We recognise that most researchers assemble figures and statistics in established tools such as Adobe Illustrator, PowerPoint, GraphPad or BioRender, and that these workflows are deeply ingrained. Rxiv-Maker is designed to sit alongside these tools, not replace them: figures prepared in any of these tools can be dropped in as static images, while the optional code-driven generation is available for authors who want their figures and reported values to update automatically with the analysis.

For cell biologists specifically, the value of the framework is best judged by how it has been used. The studies described above (Brito et al., 2026; Popović et al., 2026; Grobe et al., 2026 preprint) reported real biological results, from bacterial cell cycle regulation to filopodia function and filopodia-mediated trans-endocytosis, and the authors prepared their manuscripts with Rxiv-Maker. For groups that also adopt its optional code-driven figures, holding the text, figures and statistics in a single versioned project makes it possible to trace a reported value back to the code and data that produced it. This kind of end-to-end traceability is increasingly valued in quantitative cell biology, and it is the practical contribution Rxiv-Maker offers to the field.

Rxiv-Maker continues to evolve. We plan to integrate Pandoc (https://pandoc.org/) more deeply to enable further multi-format outputs, such as HTML and EPUB, while preserving our commitment to typographic quality. We also intend to enhance the integration of the framework with computational environment managers to further improve the portability and reproducibility of our executable manuscripts across different systems (Rubens et al., 2020; Hidalgo-Cenalmor et al., 2024), for which containerisation technologies are also a valid alternative (Boettiger, 2015; Gomes et al., 2018).

Rxiv-Maker builds on the idea of literate programming (Knuth, 1984), holding analysis code and text in one document so that a manuscript can be rebuilt from its sources and kept consistent with the analysis behind it.

## MATERIALS AND METHODS

### Rxiv-Maker framework

The Rxiv-Maker framework is implemented as a modular Python-based engine designed to ensure computational reproducibility throughout manuscript preparation. The system can produce an executable document in which figures, tables and statistical values are generated directly from source data. As previously described, Rxiv-Maker structures each project as a standard folder containing text, figures, code and configuration files, making it easy to manage with Git for transparent versioning and collaborative editing. After a manuscript is prepared, users can generate annotated ‘diff’ PDFs from the version history to review changes between milestones, similar to familiar ‘Track changes’ workflows. Rxiv-Maker is designed for local execution; Python dependencies can be installed in an isolated environment, while a fully containerised distribution is available when a pinned Python, R and LaTeX environment is required. Manuscript data is centralised in a single YAML configuration file, which defines author information, affiliations and project settings. This file can be edited directly or generated through a guided interface provided via a Jupyter or Colab notebook.

### Execution engine and manuscript conversion

The framework operates as a local-first, command line tool that compiles manuscripts quickly and provides immediate feedback. At the core of the framework is the execution engine, which identifies and runs embedded Python or R scripts within the Markdown source to generate figures and analysis outputs directly from the underlying data. Code evaluation is strictly opt-in: standard Markdown code blocks (enclosed in backticks) are rendered statically and are not executed. Code execution occurs only for explicit Rxiv-Maker execution blocks or executable figure scripts; users should review executable content before building manuscripts from untrusted sources. To maintain an efficient authoring workflow, the system implements a caching mechanism that computes content-based checksums of scripts and their data dependencies, so code is only re-executed when relevant inputs have changed. This significantly reduces rebuild times during iterative writing while preserving the integrity of the computational results. A companion Visual Studio Code extension (Fig. S3) further streamlines writing with syntax highlighting, citation autocompletion and real-time validation of configuration.

### Programmatic figure generation

Figure generation keeps a transparent and reproducible connection between the data and the final visualisations, through script-based generation with Python or R and through diagram rendering from text-based descriptions with Mermaid (Table S1; https://mermaid.js.org/). For script-based figures, .py or .R scripts are placed in the FIGURES directory and typically draw on established plotting libraries such as Matplotlib (Hunter, 2007) or Seaborn (Waskom, 2021). Rxiv-Maker executes these scripts during compilation and detects any image files they save (PNG, PDF or SVG format) for inclusion in the manuscript, so a change in the data or in the script regenerates the figure and the visualisations stay synchronised with the analysis. This method produced Figs S1 and S2.

Diagrams such as flowcharts and system architectures are described in a .mmd text file. The framework renders it into a vector or raster image with the Mermaid command line tool, which keeps diagrams under version control in the same way as code and makes them easy to modify and track over time.

### Mathematical notation

Rxiv-Maker translates Markdown-style mathematical expressions into LaTeX mathematics, so complex notation is written with familiar syntax. Inline mathematics uses single dollar delimiters (‘$...$’), which embeds expressions such as *E*=*mc*^2^ directly in the text. Display equations use double dollar signs (‘$$...$$’), which centre the expression on its own line.

The multi-pass translator protects these expressions so that conversion from Markdown to LaTeX leaves them intact. It covers mathematical and statistical notation from single symbols to multi-line equations.

### Units, concentrations and chemical formulae

Materials and Methods sections in cell biology are dense with units, concentrations and chemical names, so these must be simple to write. In Rxiv-Maker, they are typed as ordinary text and need no LaTeX. Units and symbols are written as they read: ‘Scale bar: 10 µm.’, ‘Cells were grown at 37°C’, ‘1 µg/ml DAPI’, ‘50 mM NaCl’ or ‘0.1% Triton X-100’ (a literal percent sign is written directly in the text, as in ‘5% CO_2_’). The micro sign and the Greek mu (‘μ’) are both accepted and render identically, so either form can be used, whichever the keyboard or reference manager produces. Chemical formulae and isotopes use the same subscript and superscript syntax as the rest of the document: ‘H~2~O’, ‘CaCl~2~’, ‘MgCl~2~’ and ‘10^6^ cells’ give ‘H_2_O’, ‘CaCl_2_’, ‘MgCl_2_’ and ‘10^6^ cells’, respectively. These render as true subscripts and superscripts in both the PDF and the Word export.

For authors who want the stricter typographic conventions of the siunitx and mhchem packages in LaTeX, such as fixed thin spaces between a number and its unit, the {{tex:...}} escape listed in Table S3 gives direct access to them in the PDF. The plain forms above are the recommended way to write a Materials and Methods section.

### Manuscript conversion and validation

The author's Markdown manuscript is converted into a LaTeX document using a translation engine. During the first stage, sensitive syntactic elements (mathematical equations, citation keys and code blocks) are temporarily isolated to prevent being compromised during processing. Subsequent passes normalise the document structure and map the extended Markdown syntax in Rxiv-Maker for figures, cross-references and other elements into the corresponding LaTeX commands.

The translation covers the Markdown constructs a research article needs, and Table S3 lists each construct beside the LaTeX it produces. Where a table calls for precise control over formatting, multi-column headers or mathematical notation, the {{tex:...}} escape gives direct access to LaTeX typesetting, and Table S3 was itself built that way. Text inside a {{tex:...}} block is protected from Markdown processing, so LaTeX-specific syntax passes through unchanged.

### Validation, output formats and dissemination

A multi-level validation system provides continuous quality control throughout the build process. Before compilation, the framework checks for common structural issues such as missing files or unresolved references. During compilation, LaTeX and BibTeX logs are parsed to detect errors and provide clear, actionable diagnostics. The ‘rxiv validate’ command runs these checks on demand, also reporting malformed bibliography entries, and, after compilation, it scans the PDF to flag rendering problems. In addition to generating the PDF, Rxiv-Maker supports multiple output formats. A Word export engine built on python-docx maps the same semantic Markdown source to .docx documents while preserving figure numbering, citations and equations to facilitate collaboration. Units, concentrations and chemical formulae are written as ordinary text, so a Materials and Methods section reads as it would in any word processor. For advanced formatting requirements, authors can directly inject LaTeX code using {{tex:...}} blocks (Table S3). The framework also integrates with Git to support version tracking and enables the generation of annotated PDFs with tracked changes using latexdiff.

To facilitate preprint dissemination, Rxiv-Maker provides automated commands (‘rxiv arxiv’ and ‘rxiv biorxiv’) that assemble all required submission materials. These workflows generate the manuscript PDF, supplementary files and figure assets for bioRxiv submission, or a fully compilable LaTeX source package for arXiv submission. For environmental consistency, which is particularly valuable for large collaborations or long-term archival, a fully containerised version is also available. This requires Docker (https://www.docker.com/) and encapsulates the entire build environment, so the manuscript rebuilds with the same figures and reported values on any system. The image carries pinned versions of Python, R, LaTeX and the figure-generation libraries, and is published for both x86-64 and ARM architectures, so authors on recent Apple hardware and on institutional Linux servers run the same environment. A manuscript can therefore be rebuilt without installing a LaTeX distribution at all, which removes the step that most often blocks new users.

### Preprint submission data

The arXiv monthly submission data used to generate Fig. S1 are available at https://arxiv.org/stats/monthly_submissions. Preprint submissions data across different hosting platforms (Fig. S2) are available at https://github.com/esperr/pubmed-by-year.

### Use of artificial intelligence tools

Large language model assistants, Claude (Anthropic) and open-weight models served locally with vLLM were used to edit and copy-edit the manuscript text and to help write and review source code for the Rxiv-Maker software. The authors reviewed, tested and approved all text and code, and take full responsibility for the content. No artificial intelligence tool was used to generate figures, or to collect or analyse data.

## Supplementary Material



10.1242/joces.265183_sup1 Supplementary information
