# Supplementary material for: Rxiv-Maker – an automated template engine for streamlined scientific publications
Source: J Cell Sci. 2026 Oct 12;139(19):jcs265183. doi: 10.1242/jcs.265183 (PMC13741954; doi:10.1242/jcs.265183)
Supplement: Supplementary file 1 [file joces-139-265183-s1.pdf]

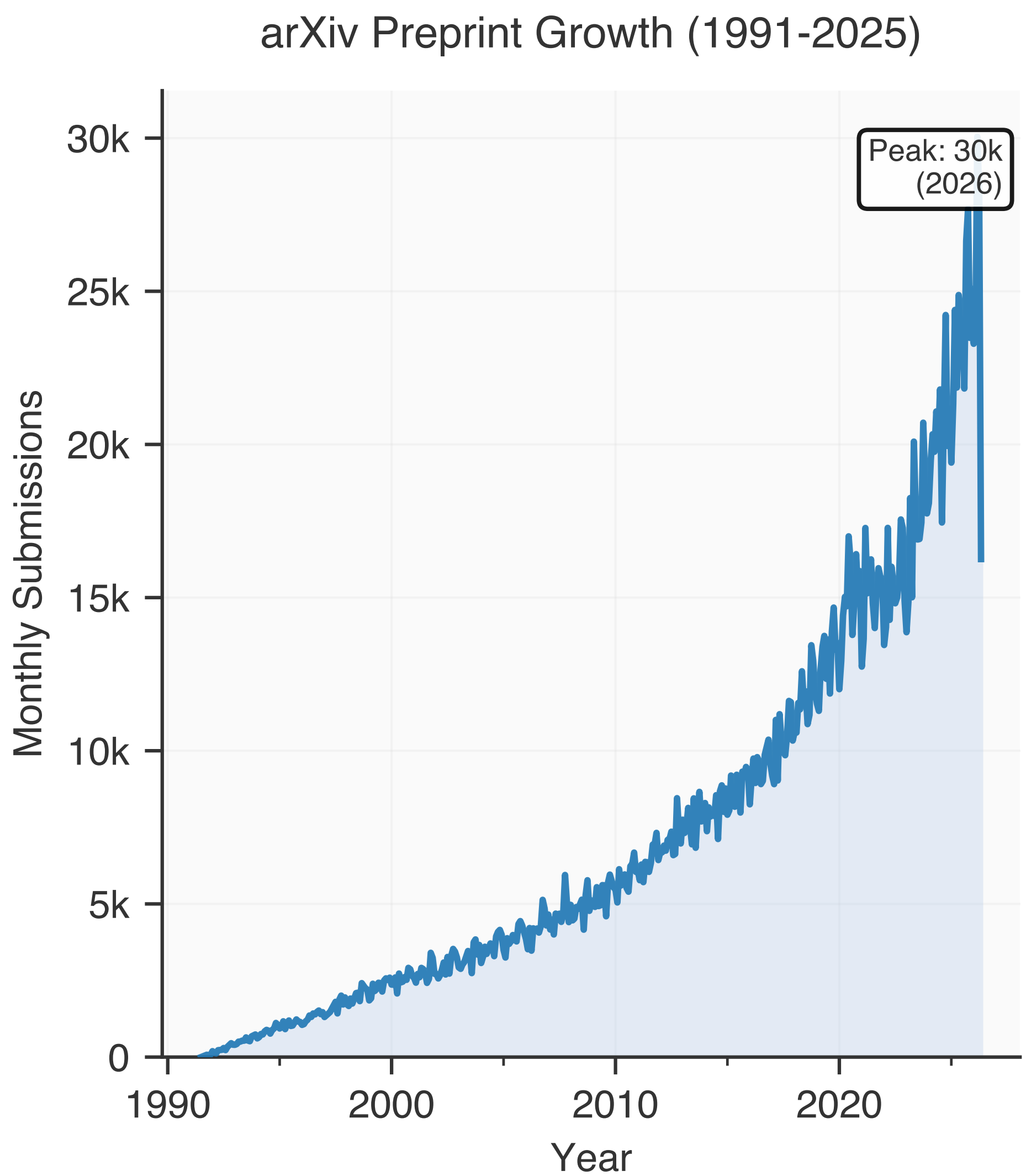

Fig. S1. The growth of preprint submissions on the arXiv server (1991-2025). This figure was generated from public arXiv statistics using a Python script executed by the Rxiv-Maker pipeline, demonstrating reproducible, data-driven visualisation.

Journal of Cell Science • Supplementary information

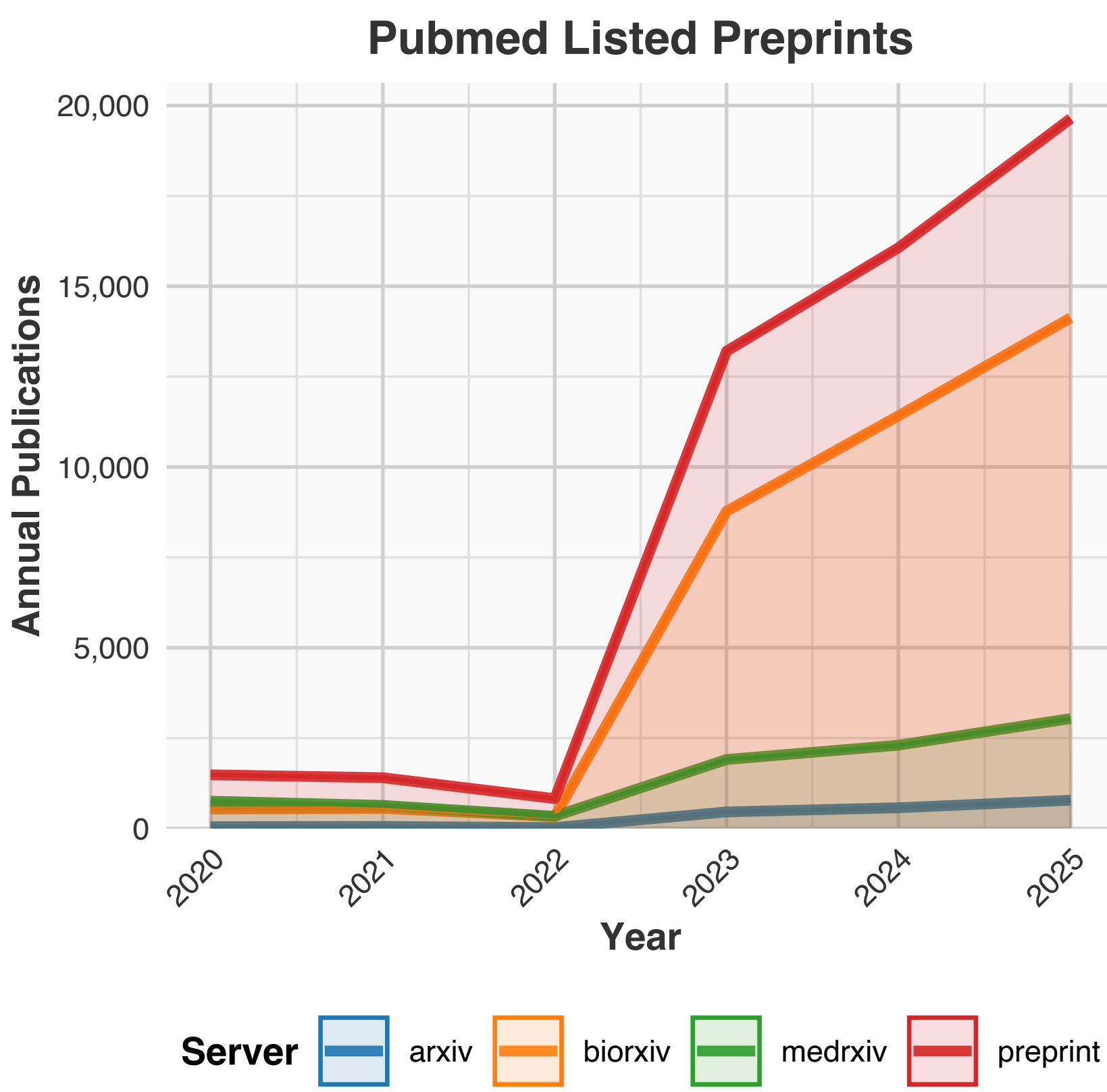

Fig. S2. Preprint Submission Trends Across Multiple Servers (2018-2025). This figure, showing preprints indexed by PubMed from major repositories, was generated from public data (https://github.com/esperr/pubmed-by-year) using a reproducible R script within the Rxiv-Maker pipeline.

Journal of Cell Science • Supplementary information

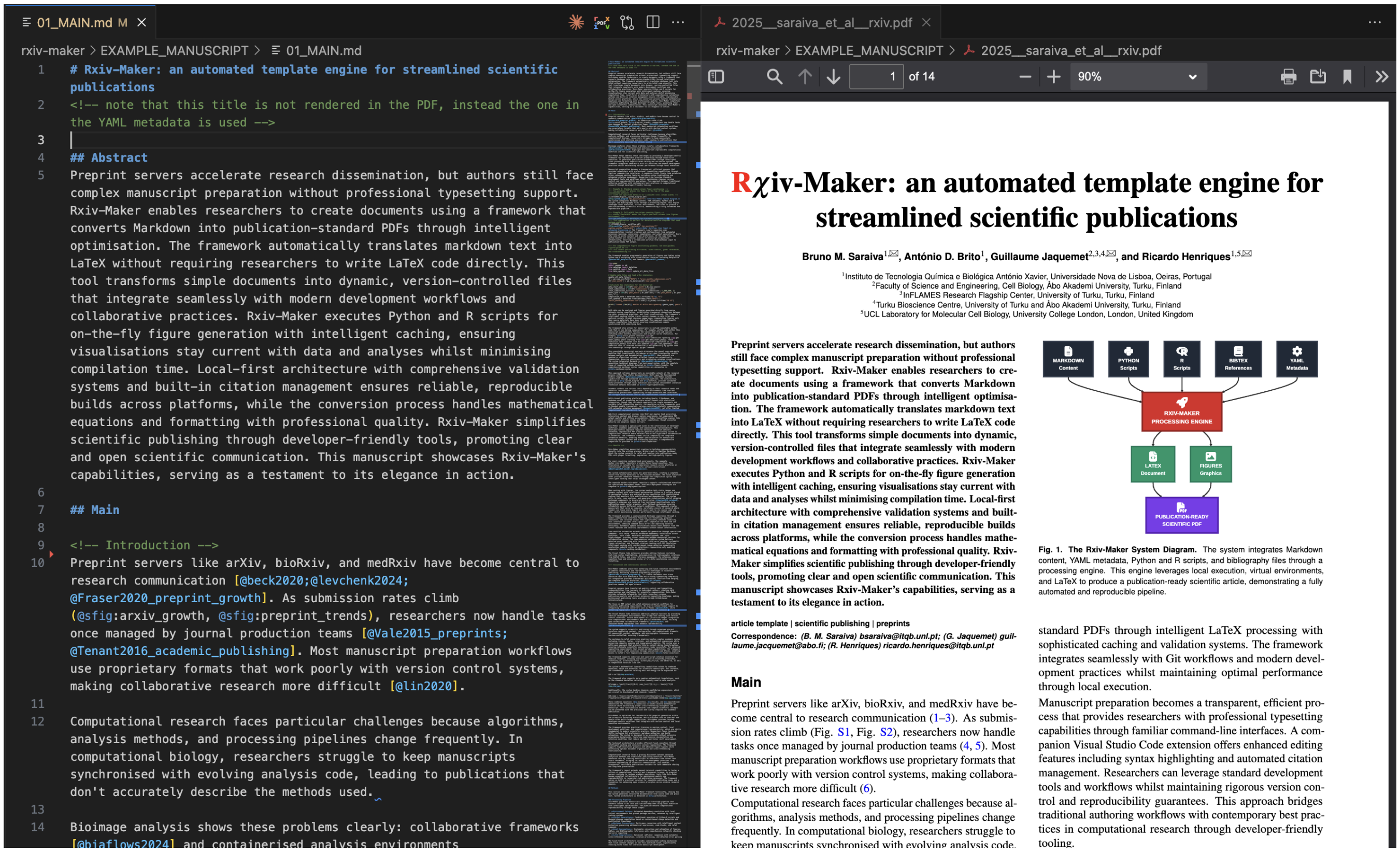

Fig. S3. The Rxiv-Maker Visual Studio Code Extension. The extension enhances the authoring experience by providing syntax highlighting for extended Markdown, autocompletion for citation keys (e.g., @Knuth...), and real-time validation to catch errors as you type.

Journal of Cell Science • Supplementary information

Table S1. Supported Figure Generation Methods. Overview of the framework's figure processing capabilities, demonstrating support for both static and dynamic content generation with an emphasis on reproducible computational graphics.

Format	Input Extension	Processing Method	Output Formats	Quality	Use Case
**Mermaid Diagrams**	`.mmd`	Mermaid CLI	SVG, PNG, PDF	Vector/Raster	Flowcharts, architectures
**Python and R Figures**	`.py`, `.R`	Script execution	PNG, PDF, SVG	Publication	Data visualisation
**Static Images**	`.png`, `.jpg`, `.svg`	Direct inclusion	Same format	Original	Photographs, logos
**LaTeX Graphics**	`.tex`, `.tikz`	LaTeX compilation	PDF	Vector	Mathematical diagrams
**Data Files**	`.csv`, `.json`, `.xlsx`	Python and R processing	Via scripts	Computed	Raw data integration

Table S2. Comparison of Manuscript Preparation Tools. The table compares tools by input format, primary use case, and workflow. Rxiv-Maker runs locally and takes Markdown as input. Overleaf supports real-time collaborative LaTeX editing, Quarto supports multiple output formats, and Manubot supports version-controlled writing.

Tool	Type	Markdown input	Primary Use Case	Main features	Open Source
**Rxiv-Maker**	Pipeline	Extended Markdown	Reproducible preprints	Local execution, caching, command-line interface	Yes
**Overleaf**	Web Editor	No (LaTeX)	Collaborative LaTeX	Real-time collaboration, templates, cloud-based	Freemium
**Quarto**	Publisher	Yes	Multi-format publishing	Polyglot support, multiple outputs, scientific focus	Yes
**Manubot**	Collaborative	Yes	Version-controlled writing	Automated citations, Git-based collaboration	Yes
**Pandoc**	Converter	Yes	Format conversion	Wide format support, extensible filters	Yes
**Typst**	Typesetter	No (Typst markup)	Modern typesetting	Fast compilation, own markup, growing ecosystem	Yes
**Bookdown**	Publisher	R Markdown	Academic books	Cross-references, multiple formats	Yes
**Direct LaTeX**	Typesetter	No	Traditional publishing	Full control, established workflows, mature ecosystem	Yes

Journal of Cell Science • Supplementary information

Table S3. Rxiv-Maker Markdown to LaTeX Translation Reference. Full mapping of Markdown syntax to the corresponding LaTeX commands.

Markdown Input	LaTeX Output	Description
**Basic Text Formatting**		
**bold text**	\textbf{bold text}	Bold formatting
italic text	\textit{italic text}	Italic formatting
__underlined text__	\underline{underlined text}	Underlined formatting for emphasis
**__bold and underlined__**	\textbf{\underline{bold and underlined}}	Nested formatting: bold containing underline
__**underlined and bold**__	\underline{\textbf{underlined and bold}}	Nested formatting: underline containing bold
__italic and underlined__	\textit{\underline{italic and underlined}}	Multiple formatting combinations
~subscript~	\textsubscript{subscript}	Subscript formatting, e.g., H_2O, CO_2
^superscript^	^{superscript}	Superscript formatting, e.g., $E=mc^2$, x^n
**Document Structure**		
# Header 1	\section{Header 1}	Top-level section
## Header 2	\subsection{Header 2}	Second-level section
### Header 3	\subsubsection{Header 3}	Third-level section
**Lists**		
- list item	\begin{itemize}\item...\end{itemize}	Unordered list
1. list item	\begin{enumerate}\item... \end{enumerate}	Ordered list
**Links and URLs**		
[link text](url)	\href{url}{link text}	Hyperlink with custom text
https://example.com	\url{https://example.com}	Bare URL
**Citations**		
@mycitation	\cite{mycitation}	Single citation
[@himmelstein2019;@Overleaf2024]	\cite{himmelstein2019,Overleaf2024}	Multiple citations
**Cross-References**		
figure:label	\ref{fig:label}	Figure reference
suppfig:label	\ref{sfig:label}	Supplementary figure
table:label	\ref{table:label}	Table reference
suptab:label	\ref{stable:label}	Supplementary table
equation:label	\eqref{eq:label}	Equation reference
note:label	\sidenote{label}	Supplement note reference
**Tables and Figures**		
Markdown table	\begin{table}...\end{table}	Automatic table formatting
Image with caption	\begin{figure}...\end{figure}	Figure with caption
**Document Control**		
<!– comment –>	% comment	LaTeX comments
<newpage>	\newpage	Manual page break
<clearpage>	\clearpage	Page break with float clearing

Journal of Cell Science • Supplementary information
