# Supplementary material for: Rxiv-Maker – an automated template engine for streamlined scientific publications
Source: J Cell Sci. 2026 Oct 12;139(19):jcs265183. doi: 10.1242/jcs.265183 (PMC13741954; doi:10.1242/jcs.265183)
Supplement: Supplementary file 2 [file joces-139-265183_review_history.pdf]

Journal of Cell Science

Rxiv-Maker – an automated template engine for streamlined scientific publications

Bruno M. Saraiva, Rita Carlota, António D. Brito, Iván Hidalgo-Cenalmor, Guillaume Jacquemet and Ricardo Henriques

DOI: 10.1242/jcs.265183

Editor: Michael Way

Review timeline

Original submission:	9 July 2026
Editorial decision:	3 August 2026
First revision received:	11 August 2026
Accepted:	11 August 2026

Original submission

First decision letter

MS ID#: jcs.265183

MS TITLE: Rxiv-Maker: an automated template engine for streamlined scientific publications

AUTHORS: Bruno M. Saraiva; Rita Carlota; António D. Brito; Iván Hidalgo-Cenalmor; Guillaume Jacquemet; Ricardo Henriques
ARTICLE TYPE: Tools and Resources

Dear Ricardo,

We have now reached a decision on the above manuscript.

As you will see, the reviewers were positive but have raised some points that will require amendments to your manuscript. Their suggestions make pretty good sense to me. I hope that you will be able to carry these out because I would like to be able to accept your paper, depending on further comments from reviewers.

Reviewer 1

Saraiva et al describe a tool (rxiv-maker) to generate preprint-ready scientific manuscripts.

It's a neat idea to be able to just write in markdown and have the package render a nicely formatted preprint. It lowers the entry-level substantially and allows the latest analyses to be included directly. There are similar tools and workflows out there, so its not exactly a new idea, but there is a chance that the community will take to this as a way of working and that is a good thing.

I'm never sure with software papers whether I am reviewing the paper, the software or both but I will give my appraisal and thoughts. Generally, I thought the manuscript and supplementary info was fine. It's a bit odd because the results and discussion sections don't really lend themselves well

© 2026. Published by The Company of Biologists under the terms of the Creative Commons Attribution License (https://creativecommons.org/licenses/by/4.0/).

to this type of paper, plus the writing is more of an advertisement than a critical piece of scientific writing you'd find in JCS!

To check the ease of use, I installed rxiv-maker with brew on macOS and had a little poke around of the output. All was OK and easy to follow. Note that on page 14 line 32 the authors should point the reader to the installation guide because opening a terminal and running the command `rxiv init my_new_paper` will do nothing. Just thinking of the complete novice reader here. The VS Code extension worked for me and was fine to install.

The authors use a non-standard way of writing md and TeX in the example manuscript. I was taught to use one line per sentence for both as the "correct" way (and two CRs for a new paragraph). This is so that when you diff e.g. during version control, the changes in the text are easier to see, and more apparent than just "there's a change somewhere in this paragraph". I'd suggest the authors adopt this approach.

How do the authors recommended that users deal with units and chemical entities? One of the wonderful things about LaTeX is that {mchem} and {siunitx} will deal with proper typesetting of concentrations, times, temperatures and common salts used in buffers etc. It seems hugely cumbersome to have to write `Scale bar, {{tex:\SI{10}{\micro\metre}}}.` in every figure legend. Since this is a paper for a cell biology journal, how do the authors recommend that cell biologists write their methods sections?

line 42 "you would write [29]." this should be the command and not the output. I noticed that the command is rendered correctly in the preprint which is up on ArXiv and on the example test pdf. Not sure what went wrong here. Just on this point, is there non-evaluation of code examples? It looks like the example code is displayed via backticks in md. Generally, TeX engines shouldn't run code (because it might be malicious) by default. In rmd/quarto `eval=FALSE` is a possibility for a block that shouldn't be run. Is this specific to referencing or does there need to be other safeguarding in place to prevent code running on collaborators' computers when they render the PDF. Having written this comment earlier, I now realise the problem is with the docx generation and not PDF, and I guess is related only to bib references being rendered by the engine. It's unlikely that a user will need to do have a raw reference in their document but nonetheless, perhaps code evaluation should be checked for both engines?

Minor comments

This link https://rxiv-maker.henriqueslab.org/getting-started/guides/ was dead at the foot of https://rxiv-maker.henriqueslab.org/getting-started/first-manuscript

page 2 line 12 "It also works smoothly with version control tools such as GitHub," GitHub is not a tool. Git is the tool (as stated correctly in the abstract).

Hey this is very minor but \color{red}R doesn't match the R in bioRxiv nor the colours of ArXiv.

<u>Reviewer 2</u>

Rxiv-Maker presented in the manuscript addresses an important need for reproducible and collaborative scientific writing by providing a framework that integrates manuscript preparation, code execution, figure generation, and version control within a single workflow. The manuscript is generally well written, the figures are clear and informative, and the authors provide convincing examples of real-world adoption through citations and use cases.

Minor comments and suggestions:

1) The manuscript would benefit from a more explicit discussion of containerization. While Docker resources appear to be available through the project ecosystem, this aspect is only briefly mentioned (or not sufficiently emphasized) in the manuscript. Containerized workflows are increasingly important for computational reproducibility because they allow users to recreate

© 2026. Published by The Company of Biologists under the terms of the Creative Commons Attribution License (https://creativecommons.org/licenses/by/4.0/).

identical software environments across operating systems and institutions. Highlighting this capability would strengthen the reproducibility message of the paper.

2) The data and code availability statements are adequate; however, I strongly recommend depositing versioned releases of both software and associated example datasets in a long-term archival repository such as Zenodo. GitHub is an excellent platform for development, but repository contents may evolve over time. Archiving specific releases in Zenodo would provide persistent DOIs, support formal citation, and ensure that readers can access the precise version associated with the publication.

3) One particularly attractive feature visible from the project resources is the possibility of testing the workflow using Google Colab. This lowers the barrier to entry for users who may be hesitant to perform a full local installation. I recommend explicitly mentioning this option in the manuscript, as it could significantly facilitate adoption among less computationally experienced users.

4) The project appears to provide extensive online documentation and tutorials. As these resources are likely to be highly valuable for new users, the manuscript could reference (on top of what is already provided in the supplementary materials) them more prominently and direct readers to installation, configuration, and example workflows.

I enjoyed reading this manuscript and believe the tool will be useful to many researchers in the biological sciences. The paper is already strong in its current form; the suggested revisions are relatively minor and mainly aim to improve discoverability of existing functionality and reinforce best practices for software preservation and reproducible research.

Overall, I find this to be a valuable and timely contribution. The manuscript is well written, the software appears mature and useful, and the presented examples adequately demonstrate its applicability. I support publication of the manuscript in its current form. The comments above should therefore be considered constructive suggestions rather than requirements for acceptance, and I believe their incorporation would further enhance the impact, usability, and reproducibility aspects of the work.

First revision

Author response to reviewers' comments

Response to Reviewers: jcs.265183

Manuscript Title: Rxiv-Maker: an automated template engine for streamlined scientific publications Authors: Bruno M. Saraiva, Rita Carlota, António D. Brito, Iván Hidalgo-Cenalmor, Guillaume Jacquemet, Ricardo Henriques

We thank the Handling Editor and both reviewers for their constructive evaluation and helpful suggestions. Below is our point-by-point response detailing the revisions made to the manuscript and the software framework.

Handling Editor Comments

> **Editor:** Please upload both a 'clean' version of your Word file, along with a highlighted version clearly showing where you have made changes in the revised manuscript. Please avoid using 'Track changes' in Word files as these are lost in PDF conversion. Please attend to all of the reviewers' comments.

Response: We have provided a clean Word file (.docx) and a highlighted Word file generated by

© 2026. Published by The Company of Biologists under the terms of the Creative Commons Attribution License (https://creativecommons.org/licenses/by/4.0/).

diffing the revised manuscript against the submitted version (insertions highlighted in green, deletions struck through in red). This highlighting is baked into the document runs and survives PDF conversion. We have also updated in-text citations throughout the .docx to the author-year format required by Journal of Cell Science. The completed submission checklist is included with the revision.

Reviewer 1

> **Comment 1:** It's a bit odd because the results and discussion sections don't really lend themselves well to this type of paper, plus the writing is more of an advertisement than a critical piece of scientific writing you'd find in JCS!

Response: We have toned down the language throughout the text, removing marketing descriptors ("polished", "publication-ready", "less user-friendly", "deep integration") and renaming the Results subsection from "From text to a publication-ready manuscript" to "From text to a manuscript". We added a paragraph in the Discussion detailing the tool's limitations: it is not a real-time WYSIWYG editor like Google Docs or Overleaf, it does not replace vector graphics tools like Adobe Illustrator for complex multi-panel diagrams, and local execution requires command-line familiarity or the Google Colab notebook.

> Comment 2: To check the ease of use, I installed rxiv-maker with brew on macOS and had a little poke around of the output. All was OK and easy to follow. Note that on page 14 line 32 the authors should point the reader to the installation guide because opening a terminal and running the command rxiv init my_new_paper will do nothing.

Response: We updated Supplementary Note 1 to state at the outset that Rxiv-Maker must be installed before running commands. We added pip and Homebrew installation instructions, linked the online installation guide (https://rxiv-maker.henriqueslab.org/getting-started/installation/), and noted the Google Colab option for running without a local install.

> **Comment 3:** The authors use a non-standard way of writing md and TeX in the example manuscript. I was taught to use one line per sentence for both as the "correct" way (and two CRs for a new paragraph). . . I'd suggest the authors adopt this approach.

Response: We agree that placing one sentence on each line improves version-control diffs. We updated the default manuscript template generated by rxiv init to follow this one-sentence-per-line format, added an explanatory comment inside the template source, and documented the convention with a before-and-after diff in the User Guide on the project website.

> **Comment 4:** How do the authors recommended that users deal with units and chemical entities? One of the wonderful things about LaTeX is that {mchem} and {siunitx} will deal with proper typesetting. . . It seems hugely cumbersome to have to write Scale bar, {{tex:\SI{10}{\micro\metre}}}. in every figure legend.

Response: In Rxiv-Maker, units, concentrations, and chemical formulas are written as plain text without LaTeX macros. Symbols like μm and °C, subscripts like H~2~O, CaCl~2~, CO~2~, and superscripts like 10^6^ are typed directly and render as true subscripts and superscripts in both PDF and Word exports. We added Supplementary Note 2 ("Units, Concentrations, and Chemical Formulae") detailing this plain-text workflow, and added a pointer in the main text. The {{tex:...}} escape hatch remains available for authors who want siunitx or mchem formatting in LaTeX PDFs, but is not required for general use.

> **Comment 5:** line 42 "you would write [29]." this should be the command and not the output. I noticed that the command is rendered correctly in the preprint which is up on ArXiv and on the example test pdf.

© 2026. Published by The Company of Biologists under the terms of the Creative Commons Attribution License (https://creativecommons.org/licenses/by/4.0/).

Response: This was caused by an exporter bug where citation syntax inside code spans was processed during Word export. We fixed this in Rxiv-Maker version 1.22.3 by protecting code spans during citation replacement. The tutorial line in the Word file now displays the literal citation syntax `[@Knuth1984_literate_programming]` instead of the rendered reference number.

> **Comment 6:** Is there non-evaluation of code examples? It looks like the example code is displayed via backticks in md. Generally, TeX engines shouldn't run code (because it might be malicious) by default. Is this specific to referencing or does there need to be other safeguarding in place. . . ?

Response: Standard Markdown code blocks (enclosed in backticks) are rendered statically as typesetting elements and are never executed by the build engine. Code evaluation is strictly opt-in and restricted to Python or R scripts placed in the FIGURES/ folder or explicitly marked {{py:...}} blocks. We added a clarifying sentence in the Methods section ("Execution engine and manuscript conversion").

> **Comment 7:** This link https://rxiv-maker.henriqueslab.org/getting-started/guides/ was dead at the foot of https://rxiv-maker.henriqueslab.org/getting-started/first-manuscript

Response: We fixed link resolution on the documentation website. Relative section links on non-index pages were resolving incorrectly under MkDocs directory URLs; we updated the documentation source to point to index files (../guides/index.md), fixing ten broken links across the site. The manuscript text link was also updated to point to the website homepage (https://rxiv-maker.henriqueslab.org).

> **Comment 8:** page 2 line 12 "It also works smoothly with version control tools such as GitHub," GitHub is not a tool. Git is the tool (as stated correctly in the abstract).

Response: We corrected the text to state that Rxiv-Maker works smoothly with Git, the version control tool, and with hosting platforms built on it such as GitHub.

> **Comment 9:** Hey this is very minor but R doesn't match the R in bioRxiv nor the colours of ArXiv.

Response: We updated the logo asset across the project repository and website, replacing the default pure red (#F80000) with a desaturated crimson (#B31B1B) matching the arXiv palette.

Reviewer 2

> **Comment 1:** The manuscript would benefit from a more explicit discussion of containerization. . . Containerized workflows are increasingly important for computational reproducibility. . .

Response: We expanded the discussion of containerisation in both the Results and Methods sections. In Results, we added a paragraph explaining why self-updating manuscripts benefit from pinned execution environments and how the Rxiv-Maker container image reproduces the same figure and numerical outputs across operating systems. In Methods, we added details on multi-architecture support (x86-64 and ARM) and noted that containerised builds allow manuscripts to be recompiled without installing a local LaTeX distribution.

> **Comment 2:** The data and code availability statements are adequate; however, I strongly recommend depositing versioned releases of both software and associated example datasets in a long-term archival repository such as Zenodo.

Response: We re-enabled the GitHub-Zenodo integration for the Rxiv-Maker repository, added .zenodo.json and CITATION.cff metadata files, and archived release v1.23.2 under the version-

© 2026. Published by The Company of Biologists under the terms of the Creative Commons Attribution License (https://creativecommons.org/licenses/by/4.0/).

independent concept DOI 10.5281/zenodo.15752358. We updated the Code Availability section to cite this concept DOI.

> **Comment 3:** One particularly attractive feature visible from the project resources is the possibility of testing the workflow using Google Colab. . . I recommend explicitly mentioning this option in the manuscript. . .

Response: We added a description of the Google Colab notebook to the Results sec- tion, including a direct link (https://colab.research.google.com/github/HenriquesLab/rxiv-maker/blob/main/notebooks/rxiv_maker_colab.ipynb) for building example manuscripts in the browser without a local installation.

> **Comment 4:** The project appears to provide extensive online documentation and tutorials. . . the manuscript could reference them more prominently and direct readers to installation, configuration, and example workflows.

Response: We updated the main text in Results to prominently introduce the project web- site (https://rxiv-maker.henriqueslab.org), directing readers to the installation instructions, first-manuscript guide, worked examples, and complete syntax reference.

Second decision letter

MS ID#: jcs.265183R1

MS Title: Rxiv-Maker: an automated template engine for streamlined scientific publications

Authors: Bruno M. Saraiva; Rita Carlota; António D. Brito; Iván Hidalgo-Cenalmor; Guillaume Jacquemet; Ricardo Henriques
Article Type: Tools and Resources

Dear Ricardo,

I am happy to tell you that your manuscript has been accepted for publication in Journal of Cell Science, pending standard publication integrity checks.

© 2026. Published by The Company of Biologists under the terms of the Creative Commons Attribution License (https://creativecommons.org/licenses/by/4.0/).
